# Factors affecting timely breast cancer treatment among black women in a high-risk urban community: a qualitative study

**DOI:** 10.1186/s12905-022-01938-0

**Published:** 2022-08-21

**Authors:** Johnie Rose, Yvonne Oliver, Paulette Sage, Weichuan Dong, Siran M. Koroukian, Sarah Koopman Gonzalez

**Affiliations:** 1 Center for Community Health Integration, Case Western Reserve University, 11000 Cedar Ave., Ste. 402, Cleveland, OH 44106-7136 USA; 2 Case Comprehensive Cancer Center, Cleveland, OH USA; 3 Freelance Qualitative Researcher, Cleveland, OH, USA; 4 Department of Population and Quantitative Health Sciences, Case Western Reserve University, Cleveland, OH USA

**Keywords:** Breast cancer, Qualitative, Disparities, Equity, Treatment delay

## Abstract

**Background:**

Black women diagnosed with breast cancer in the U.S. tend to experience significantly longer waits to begin treatment than do their white counterparts, and such treatment delay has been associated with poorer survival. We sought to identify the factors driving or mitigating treatment delay among Black women in an urban community where treatment delay is common.

**Methods:**

Applying the SaTScan method to data from Ohio’s state cancer registry, we identified the community within Cuyahoga County, Ohio (home to Cleveland) with the highest degree of breast cancer treatment delay from 2010 through 2015. We then recruited breast cancer survivors living in the target community, their family caregivers, and professionals serving breast cancer patients in this community. Participants completed semi-structured interviews focused on identifying barriers to and facilitators of timely breast cancer treatment initiation after diagnosis.

**Results:**

Factors reported to impact timely treatment fell into three primary themes: *informational*, *intrapersonal*, and *logistical*. Informational barriers included erroneous beliefs and lack of information about processes of care; intrapersonal barriers centered on mistrust, fear, and denial; while logistical barriers involved transportation and financial access, as well as patients’ own caregiving obligations. An informational facilitator was the provision of objective and understandable disease information, and a common intrapersonal facilitator was faith. Logistical facilitators included financial counseling and mechanisms to assist with Medicaid enrollment. Crosscutting these themes, and mentioned frequently, was the centrality of both patient navigators and support networks (formal and, especially, informal) as critical lifelines for overcoming barriers and leveraging facilitating factors.

**Conclusions:**

The present study describes the numerous hurdles to timely breast cancer treatment faced by Black women in a high-risk urban community. These hurdles, as well as corresponding facilitators, can be classified as informational, intrapersonal, and logistical. Observing similar results on a larger scale could inform the design of interventions and policies to reduce race-based disparities in processes of cancer care.

## Background

In 2021, an estimated 281,550 American women will have been diagnosed with breast cancer, and 43,600 will have died from the disease [1]. In the U.S., non-Hispanic white (nHw) women have the highest incidence of breast cancer among racial and ethnic groups (130.8 cases per 100,000 women), followed closely by non-Hispanic Black (nHB) women (126.7 cases per 100,000). Nonetheless, nHB female breast cancer mortality dramatically outpaces that of nHw women: 28.4 in nHB versus 20.3 in nHw, a relative difference of nearly 40% [2].

The drivers for this mortality disparity are multifactorial. Minority breast cancer patients and those of low socioeconomic status (SES) are more likely to receive their diagnosis at an advanced stage and less likely to survive their disease at any stage [1, 3]. Sixty-four percent of white women with breast cancer are diagnosed with highly-survivable localized disease, while only 55% of Black women are diagnosed at this stage [1]. At every stage, the 5-year relative survival of Black women with breast cancer is lower than that of their white counterparts [1]. Part of the explanation for this stage-specific survival disparity likely lies in the fact that Black women are twice as likely as other racial and ethnic groups to suffer from the more deadly triple negative subtype of breast cancer (seen in about 12% of all breast cancers overall) [4]. Part of the explanation, however, likely also relates to delayed treatment [5]—a key process measure in breast cancer management [3]. Numerous studies have demonstrated that Black women are more likely to experience treatment delays compared to their white counterparts [6–11], and that treatment delay is associated with poorer survival [11–14]. Most recently, Hannah and colleagues performed a meta-analysis based on five high-quality studies which showed an adjusted hazard rate of 1.08 (1.03 to 1.13 95% confidence interval) for each 4-week delay in surgical treatment of breast cancer [14].

A small number of earlier qualitative studies have provided some insight into the causes of delays in breast cancer care [15, 16]. The focus of these studies, however, has been on the causes of *diagnostic* delay; and the subjects of these studies have been breast cancer patients and survivors exclusively. With the increasing recognition of risks associated with treatment delay, we sought to learn specifically about the factors driving delays in the period from definitive diagnosis to treatment initiation. In order to gain a broader perspective, we included not only breast cancer survivors but also the caregivers or professionals serving them. To achieve the greatest potential impact on disparities in time-to-treatment, we focused on a community where risk of treatment delay is high.

## Methods

Within Cuyahoga County, Ohio (an urban/suburban county with a population of approximately 1.24 million people that is home to Cleveland), we identified a community of interest where residents face a high probability of delayed breast cancer treatment. We did so using individual-level data from the Ohio Cancer Incidence Surveillance System (OCISS), the state’s cancer registry representing women diagnosed with breast cancer from the beginning of 2010 through the end of 2015. We used SaTScan software to identify geographic hotspots based on an ordinal indicator of treatment delay, while adjusting for the underlying spatial distribution of breast cancer cases. The ordinal outcome was defined as the interval from cancer diagnosis to initial treatment, divided into categories of < 30 days, 30–60 days, and > 60 days. With this method, we defined hotspot “communities” not by census or political boundaries but by geographically defined circles encompassing no more than 5% of the population of breast cancer patients in Cuyahoga County.

Among hotspot communities, we chose the single community with the greatest degree of demonstrated treatment delay (based on the proportion of individuals experiencing > 60 days delay). From this community, we recruited breast cancer survivors or their family caregivers, and the clinical, social work, advocacy, and public health professionals serving women with breast cancer in this community. We excluded women on active treatment for breast cancer because of considerations of participant burden and the possibility of triggering added emotional stress. These participants were recruited through convenience and snowball sampling beginning with a network consisting of Community Advisory Board members from the Case Comprehensive Cancer Center (CCCC) and Community Outreach teams from CCCC-affiliated hospitals. We distributed a flier through this network, and potential participants contacted the Principal Investigator by phone or email. Participants received a $25 gift card after completing their participation. Note that recruitment was ended early due to the impact of the COVID-19 pandemic on in-person interview logistics coupled with a desire to remain consistent with our interview format.

We used a phenomenological approach [17] for developing the specific qualitative methods applied in the study, with the goal of understanding the experiences of patients, caregivers, and professionals around the phenomenon of breast cancer treatment. Between July 2019 and early March 2020, one of two qualitative researchers conducted 60–90 min, in-person, semi-structured interviews with each participant based on a detailed interview guide containing initial prompts and follow-up probing questions. The guide did not presuppose or focus on any specific possible reasons for treatment delay. Interviewers underwent identical training before beginning interviews. Interviews were transcribed verbatim and coded using NVivo software (QSR International, v12). An initial codebook was developed based on the interview guide and expected themes from the literature. A third qualitative researcher applied the codebook to the transcripts. During the coding process, emergent codes were identified and added to the initial codebook. A thematic analysis was conducted examining patterns within codes and looking at overarching themes across codes. Over the qualitative portion of the study, JR and SKG met periodically to ensure adherence to the interview guide and to identify and discuss themes around barriers to or facilitators of timely treatment from the interviews.

Both interviewers were older than 65 years of age, both identified as female, and both held advanced degrees in social sciences. Each also had significant experience performing qualitative interviews on health-related topics with local community members. Beyond personal introductions, the interviewers did not share extensive personal or professional background information about themselves with participants.

Study activities were approved by the Institutional Review Boards of Case Western Reserve University and the Ohio Department of Health, and all methods were performed in accordance with the relevant guidelines and regulations. Counseling resources were available to any participants reporting emotional distress triggered by their participation.

## Results

Among three hotspot communities identified within Cuyahoga County, we selected the single hotspot with the highest proportion of delay beyond 60 days as our sampling frame. This community spans an approximately 1.5 km radius lying along the border of Cleveland and one of its inner ring suburbs (further location details are withheld to protect subject privacy). The cluster contained 54 women diagnosed with breast cancer from 2010 to 2015, of whom 18 waited more than 60 days for treatment (compared to an expectation that eight would wait > 60 days based on county-wide experience; *p* value 0.064). The zip code which overlaps substantially with the identified hotspot features a population that is 96% African American and 3% Hispanic, with 52% of households living below the Federal Poverty Line, and a median household income of $14,603 [18].

From our recruiting network, we identified 16 individuals for semi-structured interviews. All participants were female. Eight were breast cancer survivors (all identified as nHB), two were former caretakers of breast cancer survivors, and seven were professionals caring for or otherwise serving women with breast cancer in the community. One participant fell into the categories of both survivor and professional. Table 1 describes the participants. None of these participants reported emotional distress triggered by their participation in the study.

**Table 1:** Description of participants

	N = 16
Female	16 (100%)
*Race/ethnicity*	
Non-Hispanic black	11 (69%)
Non-Hispanic white	4 (25%)
Hispanic white	1 (5%)
Mean/median age	57/60
Role*	
Survivor	8 (50%)
Caregiver of patient/survivor	2 (13%)
Nursing	2 (13%)
Social work	2 (13%)
Health system community outreach	2 (10%)
Public health	1 (6%)

*Percentages sum to > 100% because one participant reported dual roles as a survivor and professional

Barriers to and facilitators of timely treatment fell into three primary themes: *informational*, *intrapersonal*, and *logistical*. Those specific barriers/facilitators that fell under each of these main themes are described below.

### Informational barriers

Inaccurate information, a lack of practical information about processes of care, and time spent seeking additional information can each contribute to treatment delay through different mechanisms.

#### Erroneous beliefs

A barrier mentioned by both professional and survivor participants was erroneous beliefs about treatment and about cancer in general. This included beliefs that surgical treatment of cancer can cause the disease to spread to other parts of the body. One participant described the beliefs of some in her community as follows: “Well, once they cut you open it's going to spread”. Another survivor, who had experienced another type of cancer prior to their breast cancer diagnosis shared a similarly erroneous and fatalistic belief about the survivability of cancer: “I used to believe that cancer was just an automatic death sentence”.

At the other extreme, one professional mentioned having multiple patients who do not believe that cancer is a real disease.

We’ve had patients who don’t believe in cancer. We have some almost conspiracy theories about whether it’s real or maybe cultural beliefs that contradict sort of the medical facts that we present. (Professional)

#### Lack of information about process

Some participants described a lack of clear information about the steps involved in starting and maintaining treatment as a barrier. This is typified by the following quote from one participant:

Lack of information or communication can [make it] difficult for people to get their treatment because if you don’t know, this is new to you and you don’t know about something or no one is giving you information to help ease your mind of what you’re about to go through and why and what for (Survivor)

#### Information seeking

Some participants described seeking information about treatment from other providers, both traditional and alternative, as a source of treatment delay. This was mentioned by multiple professional participants as a barrier that often negatively impacts patients. One professional participant, after recounting the case of a patient who delayed traditional treatment in order to pursue an alternative therapy, stated, “People who want alternative therapy are going to do that. So many people then come back a year later, two years later with big tumor.”

On the other hand, one survivor who sought a second opinion out of concern over the impact of cancer treatment on their comorbid conditions reported the *benefit* of this additional step, and the subsequent change in treatment, without citing any negative impacts stemming from treatment delay.

### Intrapersonal barriers

Misgivings stemming from mistrust and fear, as well as sometimes powerful denial, were cited by participants as intrapersonal barriers to timely treatment initiation.

#### Mistrust

Survivor, caregiver, and professional participants described patient mistrust as a barrier to timely treatment. This mistrust encompassed not only the processes within the healthcare system, but also the efficacy of the system generally, as reflected by the comments of one participant who described “not trusting what the outcome’s going to be, doubting” (Caregiver). Two participants described a perception among female African American patients that doctors and nurses were not motivated to help them and were not making decisions in their best interests. For example, one shared the following:

Sometimes if your insurance is not that good or if the people like the doctors or the nurses that work with the doctors are not really motivated to help you, especially if you're poor and Black, then you do slip through the cracks. (Professional/Survivor)

#### Fear

Beyond fear of pain and negative health outcomes generally, participants also described fear of disfigurement, and feeling less than whole after mastectomy, as a common barrier to timely treatment initiation.

This particular lady, we were not going to take off her breast because that would not make her whole […] and she was not going to do that. (Professional)

Some women cited fear that disfigurement would lead to abandonment by their significant other.

But some people I personally know that put off the surgery or decided, ‘Even though this cancer is aggressive, I’m not going to have a mastectomy because I’m worried about my husband leaving me’. (Professional/Survivor)

#### Denial

Denial was cited by survivor and caregiver participants as another barrier to timely treatment initiation. One comment speaks to the power of denial in decision making.

I really just acted as though it was not me. I didn’t act like it. I really believed that it was not me. I thought that they had put my name with somebody else's test. And that wasn’t true. I just carried on like I wasn’t in any pain. (Survivor)

Finally, some professionals also mentioned mental health and substance abuse challenges as compounding factors which contributed to broadly challenging psychosocial contexts for patients needing to initiate treatment.

### Logistical barriers

Logistical barriers may be thought of as the final hurdles to be cleared after a woman has overcome any informational or intrapersonal barriers affecting their willingness to receive treatment. Our respondents reported the following types of logistical barriers to receiving timely treatment.

#### Transportation

Transportation challenges were cited by professional and survivor participants as a significant barrier to receiving treatment.

…for somebody who don’t have transportation, that can be a barrier. Some people who don’t drive freeways, that can be a barrier, a reason why somebody would put off mammograms or put off going to get treatment because it may be too far for them. (Survivor)

Two professional participants also cited the cost of parking as a barrier to both initiation of and continued adherence to treatment.

#### Costs related to treatment

Another barrier stemmed from the cost of receiving treatment. Beyond the direct costs of treatment (which may be fully covered under Medicaid), the lost wages and possible job loss resulting from missed work, transportation and parking costs, and the cost of requisite child care can constitute seemingly insurmountable obstacles to treatment—as well as enormous emotional stress. One participant summed up the choice that some patients are forced to make this way:

Some people just stop because, ‘Well, can I keep a roof over my head, or can I keep my chemo?’ (Professional)

#### Prioritization of other responsibilities

An indirect, but no less significant, barrier can be the caretaking responsibilities of some patients. Multiple professionals mentioned child care responsibilities, coupled with the inability to afford professional child care, as substantial practical barriers to timely treatment initiation and adherence.

### Informational facilitators

#### Objective and understandable information

Survivors noted the value of information detailing their planned course of treatment and what they should expect at each stage. Their comments suggest the utility of clear information in reducing fear and clarifying processes of care.

Being able to have pamphlets and stuff to read to help me because all this was new to me. (Survivor)

Additionally, a comment from one participant describing a previous bout with cancer points to the importance of clarity around care processes for boosting self-efficacy.

I knew how important it was to just follow through and do everything that I needed to do step by step if I wanted to live (Survivor)

### Intrapersonal facilitators

#### Faith

Faith was commonly mentioned by professionals and survivors as a mechanism for coping with the difficulties of receiving a cancer diagnosis and of treatment generally. As such, faith can be viewed as a facilitator of not only timely treatment initiation but also adherence. In recounting her previous bout with another type of cancer, one survivor shared the following:

When I first found out […] I was ready to die. And a friend of mine said that, ‘You’ve talked to everybody about this cancer. Have you talked to God?’. And I said, ‘No’. So, I went to the altar, and I prayed. When I got up, I didn't have any more worries. (Survivor)

### Logistical facilitators

Logistical facilitators identified tended to be financial in nature.

#### Financial counseling

A participant who was both a survivor and a professional pointed to the importance of financial counselors based at hospitals or community organizations in the treatment journeys of many women.

#### Medicaid enrollment

Numerous professionals described the importance of Medicaid in assuring access to treatment for many women.

Oh my God. I don’t even know how people—if you don’t have good hospitalization, and you’re not poor enough to qualify for Medicaid, I do not know how you’re handling the 20% [Medicare coinsurance]

One professional participant also described the Breast and Cervical Cancer Project (BCCP), implemented through the Centers for Disease Control and Prevention (CDC) National Breast and Cervical Cancer Early Detection Program (NBCCEDP) [19], as a critical link for enrolling uninsured women with breast cancer in Medicaid.

#### Transportation assistance

A few professionals also mentioned resources to assist with transportation-related barriers, including parking validation or arranging for free transportation.

### The importance of patient navigation and support networks

Numerous participants described the critical role of patient navigators or support networks who functioned to assist patients with overcoming multiple barriers to treatment and provided access to facilitating factors.

#### Patient navigators

Patient navigators (including individuals trained in nursing, social work, or other fields) were cited by numerous participants as essential for overcoming informational and logistical barriers to care in particular.

When asked specifically why she thinks some women face delays in starting treatment, one survivor highlighted the importance of navigators in overcoming informational barriers:

I think it’s lack of information, just figuring out the steps. I think that's why they have breast nurse navigators. (Survivor)

One professional, whose duties included serving as a patient navigator, shared the following with regard to assisting patients facing logistical barriers:

We have patients who can’t imagine how they’re going to take time off from work, when they have that really fixed income, or who don’t have anyone to watch their children, who just can’t kind of figure out the logistics. And that’s where we try to be really helpful in doing some problem solving with them and helping connect them with resources. (Professional)

Describing a prototypical scenario of a newly diagnosed breast cancer patient, one professional who was also a survivor said

‘Well, let’s see when we have another opening. It’s three months from now’. That’s where a patient navigator comes in and like, ‘No. This is a cancer diagnosis. She can’t wait another three months to get in to see somebody to start the testing and stuff’. (Professional/Survivor)

#### Support networks

Nearly ubiquitous in our interviews was the centrality of support networks as a resource for overcoming barriers to treatment initiation and adherence. These support networks might be formal, such as support groups sponsored by hospitals or community agencies, or informal, such as family, friends, or faith community members. Participants’ comments suggested that support networks can help mitigate essentially every type of barrier described. Support groups can reduce informational barriers in particular by providing important process information or correcting misinformation. Both formal and informal support networks can help patients cope with the mistrust, denial or fear which can inhibit engagement with treatment. Similarly, they can provide practical support—through connection with programs or direct assistance—to overcome transportation, financial, or other logistical barriers. While one survivor shared that “Talking to other people that have been through this, that have survived it” empowered her during treatment, more comments highlighted the centrality of less formal support networks formed before cancer diagnosis. One professional’s statement encompassed the sentiments and experiences of multiple survivors:

Well, I think probably the number one asset that kind of helps patients cope is probably support, so having that support network whether it be family, friends. For a lot of people, that's their faith, their church support or their synagogue support or whatever they worship. So often, it’s the support network that really helps patients cope and get through it. And that support network is often providing emotional support and sometimes it’s financial support. I mean, often, it’s financial support, actually, for our patients on a fixed income. It’s not just us connecting them with the resources. (Professional)

## Discussion

We identified a Cleveland neighborhood with aberrantly high proportions of women facing delayed breast cancer treatment then conducted a series of semi-structured interviews of women connected with this neighborhood—either as residents who had survived breast cancer (all of whom were Black), or as caregivers or professionals serving breast cancer patients in the neighborhood. Our participants identified numerous barriers to timely treatment which can be categorized broadly as informational, intrapersonal, or logistical. The facilitators identified can be categorized similarly. Two resources highlighted repeatedly as either mitigating barriers or connecting to/providing facilitating factors were patient navigators from hospitals or community agencies and support networks. Support networks could consist of formal support groups—again hospital or community-based—or informal networks such as family, friends, or fellow members of a faith community.

The National Cancer Institute’s Patient Navigation Research Program (PNRP) defined patient navigation (PN) as “support and guidance offered to vulnerable persons with abnormal cancer screening or a cancer diagnosis, with the goal of overcoming barriers to timely, quality care” [20]. There is limited empirical evidence regarding the efficacy of PN programs for improving the timeliness of breast cancer treatment. That which exists is relatively remote and inconclusive [21–23], suggesting an important evidence gap. If PN programs do improve timeliness, fundamental changes will need to occur in their financing if their full implementation is to be realized, especially since navigation programs which utilize employed (as opposed to volunteer) navigators appear more likely to be effective [24]. Typically, navigation programs are either internally funded by health systems or supported through short-term grant funding [25]. To maintain an employed PN workforce in the long term will require more consistent funding streams. Evolving alternative payment models which shift risk for poor outcomes onto health systems may encourage more systems to provide this funding [25]. Key to this transition will be establishing an evidence base related to value in PN programs, as well as guidance on how to design context-specific goals and measurement to fit the population served [24].

Among breast cancer patients, social networks have been shown to fulfill informational and emotional support needs [26], relieve depressive symptoms [27–29], and even alleviate pain and other sequelae [27, 29]. Racial differences in the types of social networks considered most beneficial may inform efforts to optimize the value of these networks in specific subgroups. While Paladino and colleagues noted that a social network containing other cancer patients was highly prioritized by many white women, they did not see this same pattern among Black women [26]. Nor did Flannery and colleagues, who described the most important support relationships to Black breast cancer patients as being those involving family or family-like groups and pre-existing social institutions such as church congregations [30]. While one survivor in our study did point to the utility of speaking with other survivors during her treatment phase, the plurality of comments among our participants bear out the predominance of informal support networks as a strong facilitating force for Black breast cancer patients. This finding has implications for how more formal interventions might be designed. For instance, Nonzee suggests that PNs consider engaging “key opinion leaders in a woman’s social network” in order to provide the most meaningful services [15].

Not directly mentioned, but arguably underlying most of the informational and logistical barriers described are health system factors. The fragmented nature of the U.S. health system, from the standpoints of both supply and financing, creates cracks into which the most vulnerable can easily fall [31]. The barriers described by participants in this study depict a health system which places the onus for obtaining correct information and arranging logistics or covering treatment-related costs (direct or indirect) onto the patient or their family. This perhaps explains much of the value ascribed to navigators and support networks—safety nets which can catch patients whom the system may not well serve. A more “system-like” health system—one that earns the trust of patients by providing more uniform access to services and transparent, culturally appropriate information about process—would ideally reduce the dependence on navigators and support networks as sole lifelines to essential services.

Two previous studies applied qualitative methods to the issue of timeliness of cancer care for low-income women. Nonzee et al. [15] examined barriers and facilitators related to breast and cervical cancer screening, follow-up, and treatment in the Chicago area. The focus of participants’ comments in the paper was almost exclusively on timeliness of screening and follow-up of abnormal screening results [15]. Jerome-D’Emilia and colleagues [16] interviewed 20 low-income New Jersey women affiliated with the New Jersey Cancer Education and Early Detection Program who had been diagnosed with breast cancer. They used semi-structured interviews to examine factors affecting timeliness of diagnosis and treatment (without distinguishing between the two). The major correlates of diagnostic or treatment delay were *lack of access to healthcare*, *lack of knowledge about their disease*, and *spirituality*. These barriers fit within the themes we have identified of logistical barriers, informational barriers, and intrapersonal facilitators, respectively. Our study builds upon this earlier work by focusing exclusively on treatment delay in a neighborhood with high risk of the same. We also broaden the scope of participants to include caregivers or professionals working with breast cancer patients from the focus community, thus providing multiple perspectives. These professionals bring to the study the vicarious experiences of numerous women from the same community.

Both the chief strengths and the chief limitations of the present study relate to its study sample. We used empirical spatial analysis to identify a neighborhood within our community where breast cancer patients were most likely to suffer treatment delay. Within that community, we sampled not only breast cancer survivors, but also their caregivers and the professionals who served and cared for them. These both represent innovations relative to previous work in the field. The COVID-19 pandemic required us to stop recruitment and interviews. We believe, however, that the sample size achieved was adequate based on the methodologic work of Guest et al. [32] who concluded that six interviews were generally sufficient to achieve saturation in identifying major themes, and 12 was a sufficient number to identify most subthemes. Further reassuring was the fact that issues identified by patients/caregivers and by professionals were similar. The generalizability of results to Black women in other vulnerable U.S. communities cannot be determined, of course. Additionally, survival beyond treatment was an implicit criterion for study inclusion of patient participants. This fact may have introduced a degree of survival bias, meaning that participating survivors may have experienced better than typical outcomes.

The methods used here cannot offer quantitative insight into the prevalence of any specific factor impacting the timeliness of cancer care. To address issues of generalizability and provide greater quantitative insight, a broader-based survey covering several communities is warranted. The work we have described here can inform development of such a survey. Eventually, we hope that this line of inquiry can inform the development of interventions which can be tested prospectively.

## Conclusions

We observed that Black women in a disadvantaged urban neighborhood faced numerous barriers to timely breast cancer treatment. These barriers, as well as corresponding facilitators, can be classified as informational, intrapersonal, and logistical. Crosscutting these themes, and mentioned frequently, was the centrality of both patient navigators and support networks (formal and informal). Each appears to help patients negotiate multiple barriers and take advantage of factors which facilitate treatment. If broader-based, quantitative studies yield similar results, this could inform the development of interventions and policies to reduce unnecessary treatment delay and to enhance the utilization and utility of patient navigator programs and formal support groups. In addition, these insights could provide guidance on how best to leverage patients’ pre-existing support networks to support their care.

## Data Availability

The data generated and analyzed during the current study are not publicly available due to participant privacy concerns but are available from the corresponding author on reasonable request.

## Abbreviations

BCCP: Breast and Cervical Cancer Project
NBCCEDP: National Breast and Cervical Cancer Early Detection Program
OCISS: Ohio Cancer Incidence Surveillance System
PN: Patient navigator
PNRP: Patient Navigator Research Program
